# Machine Learning Augmented Interpretation of Chest X-rays: A Systematic Review

**DOI:** 10.3390/diagnostics13040743

**Published:** 2023-02-15

**Authors:** Hassan K. Ahmad, Michael R. Milne, Quinlan D. Buchlak, Nalan Ektas, Georgina Sanderson, Hadi Chamtie, Sajith Karunasena, Jason Chiang, Xavier Holt, Cyril H. M. Tang, Jarrel C. Y. Seah, Georgina Bottrell, Nazanin Esmaili, Peter Brotchie, Catherine Jones

**Affiliations:** 1 Annalise.ai, Sydney, NSW 2000, Australia; 2 Department of Emergency Medicine, Royal North Shore Hospital, Sydney, NSW 2065, Australia; 3 School of Medicine, University of Notre Dame Australia, Sydney, NSW 2007, Australia; 4 Department of Neurosurgery, Monash Health, Melbourne, VIC 3168, Australia; 5 Department of General Practice, University of Melbourne, Melbourne, VIC 3010, Australia; 6 Westmead Applied Research Centre, University of Sydney, Sydney, NSW 2006, Australia; 7 Department of Radiology, Alfred Health, Melbourne, VIC 3004, Australia; 8 Faculty of Engineering and Information Technology, University of Technology Sydney, Sydney, NSW 2007, Australia; 9 Department of Radiology, St Vincent’s Health Australia, Melbourne, VIC 3065, Australia; 10 I-MED Radiology Network, Brisbane, QLD 4006, Australia; 11 School of Public and Preventive Health, Monash University, Clayton, VIC 3800, Australia; 12 Department of Clinical Imaging Science, University of Sydney, Sydney, NSW 2006, Australia

**Keywords:** machine learning, chest X-ray, deep learning, radiology

## Abstract

Limitations of the chest X-ray (CXR) have resulted in attempts to create machine learning systems to assist clinicians and improve interpretation accuracy. An understanding of the capabilities and limitations of modern machine learning systems is necessary for clinicians as these tools begin to permeate practice. This systematic review aimed to provide an overview of machine learning applications designed to facilitate CXR interpretation. A systematic search strategy was executed to identify research into machine learning algorithms capable of detecting >2 radiographic findings on CXRs published between January 2020 and September 2022. Model details and study characteristics, including risk of bias and quality, were summarized. Initially, 2248 articles were retrieved, with 46 included in the final review. Published models demonstrated strong standalone performance and were typically as accurate, or more accurate, than radiologists or non-radiologist clinicians. Multiple studies demonstrated an improvement in the clinical finding classification performance of clinicians when models acted as a diagnostic assistance device. Device performance was compared with that of clinicians in 30% of studies, while effects on clinical perception and diagnosis were evaluated in 19%. Only one study was prospectively run. On average, 128,662 images were used to train and validate models. Most classified less than eight clinical findings, while the three most comprehensive models classified 54, 72, and 124 findings. This review suggests that machine learning devices designed to facilitate CXR interpretation perform strongly, improve the detection performance of clinicians, and improve the efficiency of radiology workflow. Several limitations were identified, and clinician involvement and expertise will be key to driving the safe implementation of quality CXR machine learning systems.

## 1. Introduction

Chest X-rays (CXRs) have been used as the baseline chest imaging modality for more than a century [1]. This relatively simple method of image acquisition has provided access to radiological investigation of chest pathology to almost every corner of the globe, encompassing the investigation of infection, cardiac pathology, chest trauma, and malignancy. The development of safe principles of ionizing radiation usage and advancements in the acquisition of digital images have led to reduced radiation exposure, improved image quality, and wider CXR availability. The CXR remains the most frequently performed medical imaging investigation worldwide [2].

There are, however, limitations to the diagnostic utility of the CXR. Soft tissue contrast assessment is limited by the projection of X-rays through multiple organs and the generation of a two-dimensional image with superimposed densities, which can lead to reduced sensitivity for subtle findings [3]. This makes CXR interpretation particularly challenging and, as a result, most cases of missed lung cancer appear to be due to errors in CXR interpretation [4]. Human error, reader inexperience, fatigue, and interruptions contribute to interpretation inaccuracy [3,5], and the availability of experienced thoracic radiologists is limited. Other imaging modalities are capable of providing high-sensitivity visualizations of the chest, including computed tomography (CT) and ultrasound. These modalities have been shown to have higher sensitivity for many findings, including pneumothorax [6], pneumonia [7], and lung nodules [8]. However, due to widespread availability, short scan time, low cost, and low radiation exposure, the CXR remains the first line of imaging modality for chest assessment [9]. For these reasons, there have been many attempts to create artificial intelligence (AI) systems to assist radiologists in the task of CXR interpretation [10,11].

Machine learning, a subdomain of AI that involves learning patterns in data to enable effective prediction and classification, is profoundly influencing care delivery across medical specialties from pathology to radiology [12,13,14,15,16]. Deep learning image processing algorithms are based on convolutional neural networks (CNNs) and have been trained to detect pneumothorax [17], pneumonia [18], COVID-19 [19,20,21,22,23,24], pneumoconiosis [25], tuberculosis [26], and lung cancer [27]. Models have been developed to automate lung segmentation and bone exclusion [28], identify the position of feeding tubes [29], and predict temporal changes in imaging findings [30]. While these studies have not assessed the usefulness of AI models across many findings simultaneously, they have shown that deep learning diagnostic tools can improve the classification performance of radiologists in the detection of pulmonary nodules [31], pneumoconiosis [25], pneumonia [18], emphysema [10], and pleural effusion [32]. Coupling AI models with clinicians can result in higher diagnostic accuracy performance than either AI or clinicians alone [33]. In addition to this, they appear to improve reporting efficiency by reducing interpretation time [18].

Most deep learning systems developed to date, however, have been limited in scope, often to a single or a few findings [10,34]. While demonstrating high performance within their narrow application domains, their lack of clinical breadth may limit their utility in practice. Concerns have also been raised regarding potential risks and biases that may accompany the use of deep learning systems for image interpretation assistance, such as poor generalizability across populations [35] and automation bias [36].

The application of machine learning on chest X-rays to assist in the diagnosis of COVID-19 was a real-world example that highlighted both the benefits and pitfalls of medical imaging AI. Multiple algorithms have been developed for this purpose in recent years and have demonstrated high levels of accuracy in standalone tests [19,20,21,22,23,24]. However, the performance of some COVID-19 machine learning models has been shown to suffer when applied to datasets more representative of real-world cohorts [37], attributed, in part at least, to the issue of hidden stratification and confounded training data. A broad understanding of the modern capabilities of AI systems applied to CXR interpretation, as well as potential limitations, will be necessary for clinicians as these and similar tools are introduced into their workflow in the coming years. 

To that end, this literature review aimed to provide a contemporary and comprehensive overview of deep learning applications designed to facilitate CXR interpretation. Specifically, we sought to identify algorithm performance and scope, risks and benefits, and opportunities for future research and model development. Section 2 of this paper includes a description of our applied methods; Section 3, the results of our systematic review; and Section 4, a discussion of the implications of recent developments in this subdomain of applied machine learning in medicine. 

## 2. Methods and Materials

The methods applied in this systematic review were guided by the standards of the Institute of Medicine [38] and the Preferred Reporting Items for Systematic Reviews and Meta-Analysis (PRISMA) guidelines [39]. The prospective protocol was developed and approved by senior study authors. Risk of bias (ROB) within selected studies was assessed using PROBAST [40] (prediction model risk of bias assessment tool). 

### 2.1. Search Strategy

A comprehensive search strategy was developed and applied to the PubMed and ScienceDirect databases. To collate a contemporary sample of the literature within the rapidly developing field of deep learning technology, studies published between January 2020 and September 2022 were identified. The search strategy was based on combinations of domain specific and methodological search terms, both keywords and Medical Subject Headings (MeSH) terms (Table 1).

### 2.2. Eligibility Criteria

Publications were selected for full text review if they satisfied inclusion criteria: original research published in a peer review journal; published in English; involved the application of machine learning techniques to facilitate CXR interpretation and diagnosis; involved the use of CXR image data; addressed multiple radiological findings relevant to CXRs (>2 pathologies); and included data from adult patients. Studies were included if they evaluated model performance with one or more of the following performance metrics: accuracy, area under the receiver operating characteristics curve (AUC), sensitivity, specificity, positive predictive value (PPV), negative predictive value (NPV), F1 score, and Matthews correlation coefficient (MCC). Articles were excluded if they were review articles, books, book chapters, or conference abstracts; did not involve the deployment of deep learning; did not involve the processing of CXR data; focused on a nonclinical application; or focused on data from a pediatric population.

### 2.3. Study Selection Process

Database searches were completed by one author, with all references imported and consolidated into a web-based bibliographic software package (Paperpile LLC, MA, USA). Citations and study details, including abstracts, were exported to a custom excel spreadsheet for data management. Keywords (e.g., “letter”, “proceedings”, “review”, “computed tomography” (also “CT”), “pediatric”), were used to identify articles for exclusion. Duplicates were removed. Multiple authors (M.R.M, Q.D.B, H.K.A, N.E, G.S, H.C) conducted manual screening to exclude titles and abstracts that did not meet predefined eligibility criteria. Two review authors (M.R.M, J.C) repeated this manual screening review on a 20% sample of the identified studies as a quality check. There was no disagreement between the main review process and the quality check. Studies passing the title and abstract screen underwent full text review and were appraised for inclusion. Disagreement or uncertainty regarding the inclusion of an article was resolved via discussion within the review team.

### 2.4. Data Extraction and Appraisal

For each included study, specific items for data extraction were collected and coded. These included study identifiers, study characteristics such as purpose, design, type, and setting, study outcomes and performance measurement, methods for model development and validation, machine learning algorithm characteristics, study results and findings, whether the article was a duplicate, whether the article was included or excluded, and the reason for exclusion. Included studies underwent an assessment of design and methodological quality using criteria defined in Table 3 and the PROBAST [40] tool.

### 2.5. Synthesis and Assessment

A PRISMA flow diagram [41] was produced to illustrate study screening, selection, and inclusion. Study and model details, including the number of clinical findings classified, design, dataset size, datasets used, validation techniques applied, performance metrics, and key findings were tabulated to facilitate analysis and benchmarking. Outcomes and key themes were summarized using descriptive statistics.

## 3. Results

### 3.1. Included Articles

**Figure 1:**
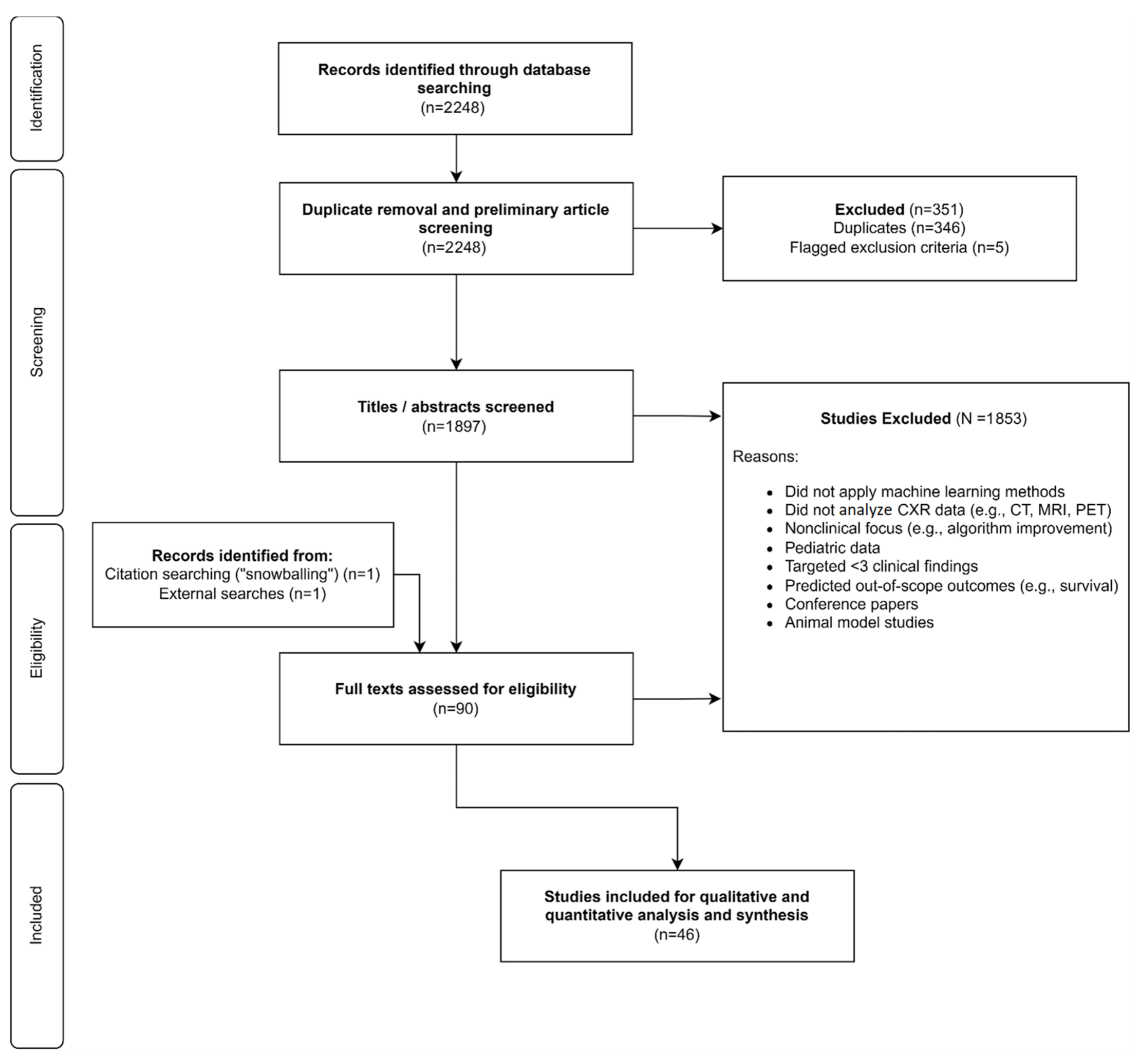
PRISMA flow diagram indicating study identification, selection, and inclusion.

The search resulted in the retrieval of 2248 records (Figure 1). We assessed 90 full text articles and included 46 in the final quantitative and qualitative analysis. Model and study details, along with ROB and quality, were summarized for each study.

### 3.2. Summary of Included Articles

The literature review identified 46 primary studies that met inclusion criteria. Most studies employed a retrospective data analysis approach to investigate device performance (97%). Only one was conducted as a prospective study in a real-world environment (Jones et al., 2021 [42]). Device performance was compared with that of physicians in 14 of the 46 included studies (30%). Of these 14 studies, device augmentation effects on clinical perception and diagnosis were evaluated in 9 out of 14 of these studies (19%). A summary of included studies, their aims, design, datasets, and number of findings identified are outlined in Table 2.

### 3.3. Quality Appraisal and Risk of Bias

Included studies underwent a quality appraisal. Results of the assessment of study quality, including appraisal criteria and scores for article quality, are presented in Table 3. The quality of studies varied across assessment domains, with some studies demonstrating a marked lack of methodological quality. A total of 29 studies were considered high quality, with an overall quality score of 70, while 15 studies were considered moderate quality, with a score of 50–60. Two studies were low quality, with an overall score of 30–40. The most common factor adversely affecting study quality was the lack of an appropriate comparator for device performance. All studies demonstrated an appropriate design, whereas only 67% involved the use of appropriate comparators. Some studies involved training a single model and did not compare its performance to other baseline models or to clinicians. Model training datasets were of sufficient size and quality in 78% of studies. Likewise, appropriate validation methods were applied in 78% of studies. Often, training dataset characteristics and validation methods were not reported; however, this was not considered a negative indicator of study quality because several studies investigated commercial or previously established devices, and these details were reported in previous studies. Appropriate sample size, performance metrics, and statistical analysis techniques were prevalent, evident in 97%, 100%, and 93% of studies, respectively.

**Figure 2:**
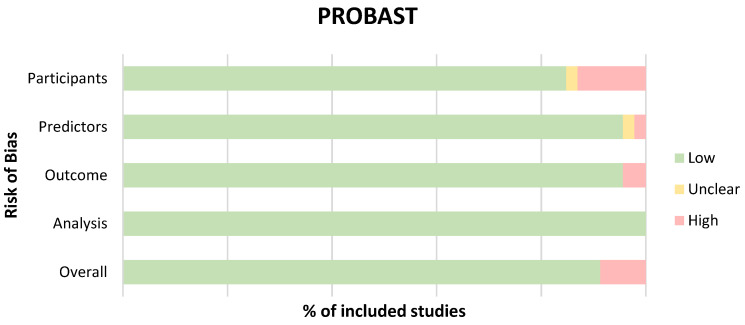
Proportion of studies with low, high, or unclear risk of bias as assessed across the four PROBAST domains.

The PROBAST [40] ROB tool assessed shortcomings in study design, conduct, and analysis that may have put the results of a study at risk of being flawed or biased. Of the 46 studies assessed, 39 were determined to be at low risk of bias, 6 at high risk, and 1 study was of unclear risk [89] (Table A1 in Appendix A). Assessment across the four PROBAST domains presented as percentages are displayed in Figure 2. The primary contributor to the high ROB in these studies was associated with patient selection methods.

### 3.4. Comprehensiveness and Algorithm Development

**Figure 3:**
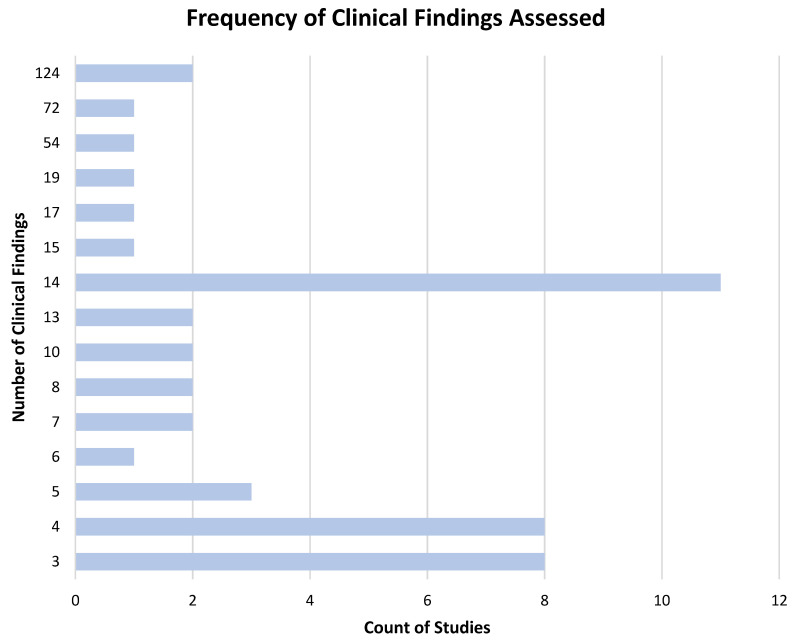
Number of findings detected across included studies.

A clear theme that emerged from this systematic review was that machine learning models designed to facilitate CXR interpretation have become substantially more clinically comprehensive. Many models were only capable of classifying less than eight clinical findings. The mean number of clinical findings classified by models was 16, with a mode of 14. The frequency of models classifying 14 findings correlates to the recurring use of the Chest X-ray14 [47] dataset, which is labeled for 14 diseases. The top three most comprehensive CXR classification models, however, markedly exceeded these benchmarks. These models were Jadhav et al., 2020 [64] with 54 findings, Wu et al., 2020 [11] with 72 findings, and Seah et al., 2021 [83] Jones et al., 2021 [42] with 124 findings. A breakdown of findings evaluated per device is presented in Figure 3. Algorithm architectures applied included UNet [96], DenseNet [97], ResNet [98], EfficientNet [99], and the VGG neural networks [100].

### 3.5. Data, Model Training, and Ground Truth Labeling

**Figure 4:**
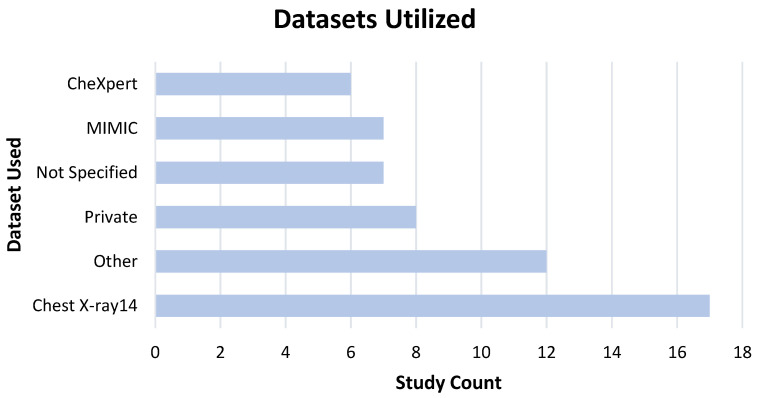
Dataset use prevalence amongst included articles.

The development of effective comprehensive CXR machine learning models relies on access to large datasets. Of the studies that reported their training and validation dataset size, on average, 128,662 images were used to train and validate models. The most comprehensive CXR models encompassing more than 10 clinical findings have been based on just four public datasets: MIMIC [65], PadChest [75], Chest X-ray14 [47], and CheXpert [52]. Figure 4 illustrates the commonly used datasets in the studies identified.

Model validation methods varied but generally adhered to the standard three-way dataset split paradigm (train–validation–test). Limited studies conducted external validation on a dataset from a different setting than the training dataset. No models were validated in a randomized controlled trial. Ground truth processes employed by researchers varied. Most studies employed a consensus of radiologists (usually two to five) who often had access to CXR reports and, in some cases, were able to correlate CXRs with CTs. A triple consensus of general (rather than subspecialist) radiologists was the most common ground truth labeling approach.

### 3.6. Performance and Safety

Identified studies used several different indicators to assess device performance, with the most common of these being the measurement of finding detection accuracy (Table 4). Comparators included other CXR models and clinician readers.

## 4. Discussion

Machine learning applied to the analysis and interpretation of CXRs carries with it significant potential for clinical quality and safety improvement. The field is developing quickly. This study was designed to comprehensively assess the performance and scope of modern algorithms and their associated risks, benefits, and development opportunities. The 46 studies included in this systematic review offer an insight into emerging themes within the contemporary landscape of deep learning models designed to interpret CXRs. There are clear trends towards increasing device comprehensiveness and improving model performance.

Published models generally demonstrated strong performance for detecting a range of clinical findings on the CXR. Some demonstrated moderate performance and likely require further development before attempts are made to apply them to clinical practice. In contrast, one comprehensive model demonstrated standout performance, with an average AUC of 0.96 across 124 findings [83]. The next most comprehensive model, which was capable of detecting 72 findings, demonstrated an average AUC of 0.77 [11]. When compared with physician detection accuracy, the identified devices were typically found to be as accurate, or more accurate, than radiologist or non-radiologist clinicians [11,43,59,63,71,74,81,82,83,88]. Taking this further, multiple studies demonstrated that use of well-trained and validated deep learning models can improve the clinical finding classification performance of clinicians when acting as a diagnostic assistance device [42,43,57,62,66,74,83,87]. This points to the potential utility and impact of machine learning systems applied to clinical practice. Transfer learning and open access to pretrained models and model architectures have underpinned the development of effective deep learning models in radiology. The continued development and optimization of these kinds of transferable models would be beneficial for facilitating further improvements in healthcare.

Another endpoint assessed by several studies was reporting and interpretation efficiency. Some included studies evaluated the performance of high-accuracy devices within the scope of developing triage or prioritization tools, which are designed to alert clinicians to cases suspected of containing time-sensitive findings. These devices have the potential to improve efficiency and patient safety by reducing the time between image acquisition and reporting by the physician. Simulation studies indicate that when these devices are used to triage studies, the report turnaround time (RTAT) of cases that include time-sensitive findings is significantly reduced [49,74]. In addition to RTAT, reporting time is another indicator used to measure efficiency. Several studies investigated the impact of AI-assisted interpretation on reading time, with some studies indicating that reporting time was reduced [43,74,87], while others found that reporting time was increased [42]. A demonstrable impact to patient outcomes may follow AI-enabled efficiency gains to radiology workflows; however, further research is necessary to establish the presence or extent of such benefits.

While the majority of studies were conducted on retrospective datasets, one study was conducted in a prospective real-world reporting environment and evaluated radiologist agreement and impact on clinical decision making due to device findings [42]. Results indicated that the radiologist and device were in complete agreement in 86.5% of cases, and device predictions led to significant report changes, changed patient management planning, and altered further imaging recommendations in 3.1%, 1.4%, and 1.0% of cases respectively. A similar retrospective study was conducted, producing comparable results [68]. In another study, a device was used to flag cases suspected of containing clinically significant findings that were initially labeled normal [62]. The device initially overlooked relevant abnormalities with a detection yield and a false referral rate of 2.4% and 14.0%, respectively.

### 4.1. Risk and Safety

Several recurring risks were highlighted by researchers including the potential for poor model generalizability, suboptimal case labeling, and the potential for data perturbation. The overfitting of CXR models has also been identified as a performance risk, leading to overestimation of performance or poor generalizability of machine learning models on external datasets [9]. External validation is an important issue in applied machine learning that has potential implications for patient safety. Some evidence suggested that high performing models may not generalize well [69]. In this review, only a limited number of included studies performed external validation of the evaluated device. Some studies reported significant drops in model performance when they were applied to external data [73,85]. These studies that reveal the so called ‘generalization gap’ underscore the need for vigilance by healthcare providers whenever efforts are made to translate machine learning models into clinical practice.

Limitations in availability of large, high quality, and accurately labeled CXR datasets can present a potential risk for developing and testing high performing and appropriately generalizable machine learning models [9]. More than half of included studies used training data from publicly available datasets originating solely from US patients (Chest X-ray14 [47], CheXpert [52], MIMIC [65]), while many others used curated private datasets with images from institutions limited to a single country or region [42,55,56,59,62,66,68,74,76,77,81,87,88]. A limited number of studies leveraged data from multiple countries [44,46,82,83,84,95]. Additional generalizability studies are required to test and verify the performance of deep learning models across different patient populations. The ethical public release of large de-identified datasets may facilitate the development of higher quality and more generalizable machine learning systems.

Natural language processing (NLP) can be problematic and noisy when used for the generation of training or ground truth labels [53]. At present, several common public datasets use NLP on the original radiologist reports to identify pathology contained in CXR images [47]. Reports are often incomplete representations of clinical findings present in the associated imaging. NLP is, therefore, prone to inaccurate image annotation, leading to negative downstream effects on model training. For example, it has been reported that the NLP-generated labels in the ChestX-ray14 [47] dataset, which was used in 17 studies, do not accurately reflect the visual content of the CXR images [101]. Investments in high-quality data labeling by expert clinicians may serve to address this issue, but these activities are resource intensive.

Testing datasets should ideally be representative of the target population (e.g., include diverse demographic groups) and the target disease, condition, or abnormality for which the model is intended. The use of datasets that include limited patient subgroups or are enriched for particular findings may not reflect the true prevalence of a disease or condition in the real world, potentially leading to spectrum bias. Spectrum bias present in the dataset can lead to model generalizability issues, resulting in reduced performance and limited clinical applicability. Several studies were identified that may have been affected. Examples include datasets that contained only one or two findings per image [57,87], only included CXRs with an associated follow-up CT scan [66], and datasets that were hand-picked rather than consecutively selected [81,82]. Further work testing and demonstrating the generalizability characteristics of published models is warranted and will serve to reinforce user confidence and patient safety.

Another consideration is the potential negative influence of AI systems on physician decision making. Automation bias, where overreliance on automated systems may lead to false positives being overlooked or a reluctance to question the suggestions made by the AI model, appears to be a particular risk for less experienced clinicians. While these issues were not assessed empirically in the included studies, the issue was highlighted and discussed. Models with a high false positive rate may require greater clinical expertise to separate true from false positives [61,62,67]. Conversely, evidence also suggests that less experienced clinicians may see the greatest benefit from AI diagnostic assistance [42]. To mitigate the risk of automation bias, manufacturers are expected to clearly report the performance details of their AI assist devices, and clinicians are expected to understand the performance characteristics and limitations of the systems they use. When developing algorithms for real-world use, vendors should be aware of evolving evidence pertaining to the mitigation of automation bias, including implementation principles and interface design choices [102].

The quality of the dataset labeling method is likely to be a cornerstone of safe deep learning model development for systems intended for clinical use. Open source datasets may be vulnerable to adversarial perturbation, which can induce model failure or falsely high performance in image classification tasks [103]. Image perturbations are often difficult to detect. They can be extremely small (a few pixels) and hence may not substantially affect data distributions. Attention to data security controls is necessary for systems intended for clinical application.

AI-assisted triage may lead to longer RTAT in the case of false negatives through down-prioritization of these cases and up-prioritization of cases with positive AI predictions. One study highlighted that there was a risk of false negatives leading to greatly increased RTAT for these studies, which would equate to a significant delay in patient treatment in the real world [49]. The performance characteristics and limitations of clinically applied models must be rigorously evaluated and clearly understood.

### 4.2. Benefits

The clinical benefits of AI models for medical image interpretation can be divided into two primary domains: improved accuracy in detecting pathology on the image, and improved reporting efficiency. Improved reporting accuracy was highlighted in numerous included studies [42,43,57,62,66,74,83,87]. This has the potential to reduce false positive and false negative rates and reduce unnecessary follow-up CT examinations and associated radiation exposure. This may lead to earlier finding detection and improved patient outcomes in screening, outpatient, emergency, and inpatient settings. While the majority of studies appeared to demonstrate improved physician performance with diagnostic device assistance, the device evaluated by Hwang and colleagues focused on detecting false negatives in CXRs originally interpreted as normal by radiologists [62]. CXRs with “normal” reports were assessed by the AI model. Researchers demonstrated a false referral rate of 0.97% and found that 1.2% contained salient clinical findings. Employing machine learning models to reduce false negative rates and improve the quality of reporting in this way will continue to be of interest to radiology providers as workload volume and complexity grow.

Several included studies demonstrated improved reporting efficiency, which coalesced into two primary categories. These were (1) reduced time to report studies that contain critical pathology [49,74] and (2) a reduction in reporting time per case [43,74,87]. An increase in reporting efficiency may impact patient outcomes by reducing the time to treatment for patients presenting with time-sensitive pathologies and increasing the rate at which physicians can report CXRs.

A further benefit identified was the ability for some AI models to provide consistent detection accuracy across variations in image quality. Some studies demonstrated that model performance was resilient to different image sources and suboptimal acquisition quality [57,60,82,88], demonstrating this kind of model resilience provides additional quality and safety assurance to the practicing clinician.

In addition to the benefits outlined above, a study conducted by Jabbour and colleagues highlighted the value of using an AI model capable of combining and evaluating patient information from multiple sources to further improve diagnostic accuracy [63]. In this study, a model designed to differentiate between causes of acute respiratory failure was trained using CXRs and clinical data from electronic health records, leading to a detection accuracy similar to, or better than, clinician readers. The application of multimodal AI systems is a developing trend in medicine [104]. A summary of the clinical benefits identified in the included studies is presented in Table 5.

### 4.3. Study Strengths and Limitations

The strengths of this systematic review include adherence to the PRISMA guidelines and standards of the Institute of Medicine and a critical assessment of risk of bias for the included studies using a robust assessment tool, PROBAST. Another was the comprehensive search strategy applied and the replicated screening review by multiple authors of a portion of identified studies as a quality control process. Limitations of this review include the use of a detailed although unvalidated tool for the assessment of study quality and a restriction of our screened studies to the English language. Recent evidence suggests that an English language search strategy restriction is unlikely to affect results [105].

## 5. Conclusions

Deep learning has been widely applied to successfully facilitate CXR interpretation. Models have been developed to classify a wide range of pathologies, and it is evident that models are becoming progressively more clinically comprehensive. It is also apparent that classification performance is improving over time.

This review focused on machine learning devices for classification of CXRs, revealing that many such software devices have been developed since January 2020. The benefits of the devices described fall under several categories, including improved pathology detection accuracy, improved triage to reduce time to treatment for critical findings, and a reduction in reporting time.

While the benefits of these devices were well reported, the potential risks associated with their adoption remained poorly characterized, with risks only superficially noted in some primary studies and not examined explicitly. The key risks associated with these devices include the potential for dataset spectrum bias, resulting from datasets not being reflective of the real-world environment, potentially limiting their clinical application. Additionally, external validation to test model generalizability was often not reported. Another risk, particularly for less experienced clinicians, is automation bias.

The world is currently experiencing a global shortage of radiologists and increased rates of clinician burnout [106]. In the United States, the number of radiologists as a percentage of the physician workforce is decreasing, and the geographic distribution of radiologists favors larger, more urban settings [107]. Even when trained radiologists are available, CXRs are often interpreted first and acted upon by non-radiologist clinicians such as intensivists and emergency physicians [108]. In developing countries, radiology services are scarce. As of 2015, only 11 radiologists served the 12 million people of Rwanda, while the entire country of Liberia, with a population of four million, had only two practicing radiologists [108]. In our experience, in some health systems, as few as one in ten CXRs are ever reviewed and reported by a radiologist. The accurate automated analysis of radiographs has the potential to improve radiologist workflow efficiency and extend life-changing clinical expertise to underserved regions [49]. In developing countries, solving the cost, complexity, skill requirement, and sustainability issues of radiology services has been a long-standing challenge [109,110]. The use of deep learning diagnostic adjuncts represents potential for increasing radiology capacity and providing better access to these services for patients.

The quality of clinical machine learning decision support systems is dependent upon the quality of the full product development lifecycle, from initial design to post-implementation monitoring [111,112]. Careful data curation and processing are required to ensure that data are broadly representative of clinical populations, to manage label fidelity and to ensure quality model training and validation [71]. Robust clinical evidence is required to demonstrate reliability, validity, safety, and beneficial clinical impact. Usability and interpretability for clinical end users are critical to adoption, and effective post-implementation performance and safety monitoring is key to quality management and ensuring patient care improvement [113].

The immediate future of applied machine learning in CXRs seems likely to follow the trends established in this systematic review. Broader comprehensiveness and continual improvements in model performance will approach and exceed that of human expert counterparts. In pursuit of these aspirations, we may see increasing use of novel development techniques such as generative adversarial networks (GANs) to augment training datasets and overcome the challenge of data limitations [114]. CXR data may be drawn upon by multimodal deep learning models and combined with other modalities such as ECGs to better predict specific disease states [115,116]. Early work has even shown that two-dimensional CXRs can be used to reconstruct three-dimensional CT images and improve pathology detection and classification efforts [117]. Interpretation automation may benefit patients in communities lacking radiologist expertise and where investigations presently go unreported.

Machine learning is driving the future of radiology. Developments will require shifts in clinical practice and careful risk mitigation. Radiologists need to be a part of the machine learning development process and drive the safe implementation of high-quality systems. Radiologists will play a key role in quality control and innovation as machine learning systems are applied to achieve better patient outcomes at scale.

## Figures and Tables

**Table 1:** Search terms used in the search strategy. * Designates truncated search for variant spellings.

	Region	Modality	Methodology	Task	Performance
Keyword	chest thora * cardiorespiratory	CXR X-ray radiograph	artificial intelligence machine learning neural net * radiomics supervised learning random forest naive bayes CNN convolution *	diagnosis image interpretation radiographic image interpretation decision Support system classif * screen detect * interpret * identifi *	diagnos * prognos * inferiority validat * superiority predict * reader * decision * clinical * risk * classif * performance bootstrapping split sample area under the curve ROC AUC performance sensitiv * accura *
MeSH Terms			machine learning artificial intelligence Neural Networks, Computer		diagnosis roc curve sensitivity and specificity triage

**Table 2:** Summary of studies identified that evaluate comprehensive CXR deep learning models.

Study	Study Aim	Study Design	Datasets Used	Dataset Size	Number of Pathologies Investigated
Ahn et al., 2022 [43]	Evaluate whether a deep-learning–based AI engine used concurrently can improve reader performance and efficiency in interpreting CXR abnormalities	Retrospective reader study	Two sources: MIMIC-CXR (public) and MGH (private)	MIMIC-CXR: 247 images; MGH: 250 images	4
Albahli et al., 2021 [44]	To achieve a fast and more accurate diagnosis of COVID-19	Retrospective	COVID Chest X-ray dataset [45]	112,812	15
Altaf et al., 2021 [46]	Classify thoracic pathologies	Retrospective	Chest X-ray14 [47], COVID-19 CXRs [48]	112,777	14
Baltruschat et al., 2021 [49]	Evaluate whether smart worklist prioritization by AI can optimize radiology workflow and reduce report turnaround times (RTATs) for critical findings in CXRs	Retrospective workflow simulation study	Chest X-ray14 (public) (112,120), Open-I dataset (public) (3125)	112,120 + 3125 images	8
Bharati et al., 2020 [50]	Develop a new hybrid deep learning algorithm suitable for predicting lung disease from CXR images	Retrospective	Chest X-ray14 [47]	112,120	14
Chakravarty et al., 2020 [51]	Develop a CXR pathology classifier	Retrospective	CheXpert [52]	223,648	13
Chen et al., 2020 [53]	Present a deep hierarchical multi-label classification approach for CXRs	Retrospective	PLCO dataset [54]	198,000	19
Cho et al., 2020 [55]	Evaluate the reproducibility of CADs with a CNN on CXRs of abnormal pulmonary patterns in patients	Retrospective	-	9792	5
Cho et al., 2020 [56]	Develop a convolutional neural network to differentiate normal and five types of pulmonary abnormalities in CXRs	Retrospective	-	9534	5
Choi et al., 2021 [57]	Evaluate the deep-learning-based CAD algorithm for detecting and localizing three major thoracic abnormalities on CXRs and compare the performance of physicians with and without the assistance of the algorithm	Reader study using retrospective data	-	244	3
Fang et al., 2021 [58]	Propose a deep learning framework to explore discriminative information from lung and heart regions	Retrospective	Chest X-ray14 [47]	112,120	14
Gipson et al., 2022 [59]	Evaluate the performance of a commercially available deep CNN for detection of traumatic injuries on supine CXRs	Retrospective	Internal dataset (private)	1404 patients/images	7
Gündel et al., 2021 [60]	Train high performing CXR abnormality classifiers	Retrospective	Chest X-ray14 [47], PLCO [54]	297,541	17
Han et al., 2022 [61]	Develop ChexRadiNet to utilize radiomics features to improve abnormality classification performance	Retrospective	Chest X-ray14	112,120 images	14
Hwang et al., 2022 [62]	Investigate the efficacy of utilizing AI for the identification and correction of false-negative interpretations in consecutive CXRs that were initially read as normal by radiologists	Retrospective feasibility study	Dataset from Seoul National University Hospital (private)	4208 images	3
Jabbour et al., 2022 [63]	Validate a model to act as a diagnostic aid in the evaluation of patients with acute respiratory failure combining CXR and EHR data	Retrospective	CheXpert and MIMIC-CXR-DICOM (public)	1618 patients	3
Jadhav et al., 2020 [64]	Predict a large set of CXR findings using a deep neural network classifier and improve prediction outcomes using a knowledge-driven reasoning algorithm	Retrospective	MIMIC [65]	339,558	54
Jin et al., 2022 [66]	Evaluate a commercial AI solution on a multicenter cohort of CXRs and compare physicians’ ability to detect and localize referable thoracic abnormalities with and without AI assistance	Retrospective reader study	Dataset from respiratory outpatient clinics (private)	6006 patients/images	3
Jones et al., 2021 [42]	Evaluate the real-world usefulness of the model as a diagnostic assistance device for radiologists	Real-world prospective reader study	Internal dataset (private)	2972 cases	124
Kim et al., 2021 [67]	Test the performance of a commercial algorithm	Retrospective generalizability study	-	5887	3
Kim et al., 2022 [68]	Evaluate the concordance rate of radiologists and a commercially available AI for thoracic abnormalities in a multicenter health screening cohort	Retrospective reader study	Health screening dataset (private)	3113 patients/images	3
Kuo et al., 2021 [69]	Explore combining deep learning and smartphones for CXR-finding detection	Retrospective generalizability study	CheXpert [52], MIMIC [65]	6453	6
Lee et al., 2022 [70]	Create a model that counters the effects of memory inefficiency caused by input size and treats high class imbalance	Retrospective	ChestX-ray14 and MIMIC-CXR (public)	Training—77,871 images, Testing—25,596 + 227,827 images	14
Li et al., 2021 [71]	Investigate the performance of a deep learning approach termed lesion-aware CNN to identify 14 different thoracic diseases on CXRs	Retrospective	Chest X-ray14 [47]	10,738	14
Majkowska et al., 2020 [72]	Develop and evaluate deep learning models for CXR interpretation by using radiologist-adjudicated reference standards	Retrospective	Chest X-ray14 [47]	871,731	4
Mosquera et al., 2021 [73]	Present a deep learning method based on the fusion of different convolutional architectures that allows training with heterogeneous data with a simple implementation and evaluates its performance on independent test data	Retrospective	Chest X-ray14 [47]	5440	4
Nam et al., 2021 [74]	Develop a deep learning algorithm detecting 10 common abnormalities on CXRs and evaluate its impact on diagnostic accuracy, timeliness of reporting, and workflow efficacy	Reader study using retrospective data	PadChest [75]	146,717	10
Niehues et al., 2021 [76]	Develop and evaluate deep learning models for the identification of clinically relevant abnormalities in bedside CXRs	Retrospective	-	18,361	8
Park et al., 2020 [77]	Investigate the feasibility of a deep-learning–based detection system for multiclass lesions on CXRs, in comparison with observers	Reader study using retrospective data	-	15,809	4
Paul et al., 2021 [78]	Propose a method for few-shot diagnosis of diseases and conditions from CXRs using discriminative ensemble learning	Retrospective	Chest X-ray14 [47], Openi [79]	>112,000	14
Pham et al., 2021 [80]	Present a supervised multi-label classification framework based on CNNs for predicting the presence of 14 common thoracic diseases	Retrospective	CheXpert [52]	224,316	13
Rudolph et al., 2022 [81]	Develop an AI system that aims to mimic board-certified radiologists’ performance and support non–radiology residents in clinical settings lacking 24/7 radiology coverage	Retrospective reader study	EU CXR dataset (private)	563 images	4
Rudolph et al., 2022 [82]	Investigate multiple clinically relevant aspects that might influence algorithm performance, considering patient positioning, reference standards, and comparison to medical expert performance	Retrospective reader study	3 cohorts (private)	3 cohorts: 563 images, 6258 images, and 166 patients, respectively	7
Seah et al., 2021 [83]	Assess the accuracy of radiologists with and without the assistance of a deep learning model	Reader study using retrospective data	MIMIC [65], PadChest [75], Chest X-ray14 [47], CheXpert [52]	821,681	124
Senan et al., 2021 [84]	Introduce two deep learning models, ResNet-50 and AlexNet, to diagnose X-ray datasets collected from many sources	Retrospective	Chest X-ray dataset comprising images from several public sources	21,165 images	4
Sharma et al., 2020 [85]	Create efficient deep learning models, trained with CXR images, for rapid screening of COVID-19 patients	Retrospective	Montgomery County X-ray Set [86]	352	4
Sung et al., 2021 [87]	Evaluate effects of a deep learning system on radiologist pathology detection	Reader study using retrospective data	-	228	5
Van Beek et al., 2022 [88]	Evaluate the performance of a machine-learning-based algorithm tool for CXRs, applied to a consecutive cohort of historical clinical cases, in comparison to expert radiologists	Retrospective reader study	Internal training dataset (private) from primary care and ED settings	Training—168,056 images, Testing—1960 images	10
Verma et al., 2020 [89]	Implementation of computer-aided image analysis for identifying and discriminating tuberculosis, bacterial pneumonia, and viral pneumonia	Retrospective	Shenzhen chest X-ray set [86]	5894	3
Wang et al., 2021 [90]	Construct a multi-scale adaptive residual neural network (MARnet) to identify CXR images of lung diseases and compare MARnet with classical neural networks	Retrospective	Chest X-ray14	13,382 images	4
Wang et al., 2020 [91]	Propose a novel deep convolutional neural network called Thorax-Net to diagnose 14 thorax diseases using CXRs	Retrospective	Chest X-ray14 [47]	112,120	14
Wang et al., 2021 [92]	Propose the triple-attention learning (A 3 Net) model	Retrospective	Chest X-ray14 [47]	112,120	14
Wang et al., 2020 [93]	Use deep learning techniques to develop a multi-class CXR classifier	Retrospective	Chest X-ray14 [47]	112,120	14
Wu et al., 2020 [11]	Assess the performance of AI algorithms in realistic radiology workflows by performing an objective comparative evaluation of the preliminary reads of AP CXRs performed by an AI algorithm and radiology residents	Reader study using retrospective data	CheXpert [52], MIMIC [65]	342,126	72
Xu et al., 2020 [94]	Explore a multi-label classification algorithm for medical images to help doctors identify lesions	Retrospective	Chest X-ray14 [47]	112,120	14
Zhou et al., 2021 [95]	Develop and evaluate deep learning models for the detection and semiquantitative analysis of cardiomegaly, pneumothorax, and pleural effusion on chest radiographs	Retrospective	Montgomery County Department of Health and Human Services, Shenzhen No. 3 People’s Hospital [86]	2838	3

**Table 3:** Quality appraisal of included studies. NA, not applicable (article not disqualified if the model training or validation methods were not relevant for reporting due to the study design, e.g., MRMC studies examining CXR reader performance). For cumulative study quality score, Yes and NA = 10, No = 0.

Study	Appropriate Study Design	Appropriate Comparators	Appropriate Training Dataset	Appropriate Validation Methods	Appropriate Sample Size	Appropriate Metric Used to Measure Performance	Appropriate Statistics Methods Used to Measure Performance	Study Quality Score
Ahn et al., 2022 [43]	Yes	Yes	Yes	NA	Yes	Yes	Yes	70
Albahli et al., 2021 [44]	Yes	No	Yes	Yes	Yes	Yes	Yes	60
Altaf et al., 2021 [46]	Yes	No	Yes	Yes	Yes	Yes	Yes	60
Baltruschat et al., 2021 [49]	Yes	Yes	Yes	Yes	Yes	Yes	Yes	70
Bharati et al., 2020 [50]	Yes	No	Yes	Yes	Yes	Yes	Yes	60
Chakravarty et al., 2020 [51]	Yes	Yes	Yes	Yes	Yes	Yes	Yes	70
Chen et al., 2020 [53]	Yes	No	Yes	Yes	Yes	Yes	Yes	60
Cho et al., 2020 [55]	Yes	Yes	NA	Yes	Yes	Yes	Yes	70
Cho et al., 2020 [56]	Yes	No	Yes	Yes	Yes	Yes	Yes	60
Choi et al., 2021 [57]	Yes	Yes	NA	Yes	Yes	Yes	Yes	70
Fang et al., 2021 [58]	Yes	Yes	Yes	Yes	Yes	Yes	Yes	70
Gipson et al., 2022 [59]	Yes	Yes	Yes	NA	Yes	Yes	Yes	70
Gündel et al., 2021 [60]	Yes	Yes	Yes	Yes	Yes	Yes	Yes	70
Han et al., 2022 [61]	Yes	Yes	Yes	NA	Yes	Yes	Yes	70
Hwang et al., 2022 [62]	Yes	Yes	NA	NA	Yes	Yes	Yes	70
Jabbour et al., 2022 [63]	No	No	No	NA	Yes	Yes	No	30
Jadhav et al., 2020 [64]	Yes	No	Yes	Yes	Yes	Yes	Yes	60
Jin et al., 2022 [66]	Yes	Yes	Yes	Yes	Yes	Yes	Yes	70
Jones et al., 2021 [42]	Yes	Yes	Yes	NA	Yes	Yes	Yes	70
Kim et al., 2021 [67]	Yes	No	NA	NA	Yes	Yes	Yes	60
Kim et al., 2022 [68]	Yes	Yes	Yes	NA	Yes	Yes	Yes	70
Kuo et al., 2021 [69]	Yes	Yes	NA	Yes	Yes	Yes	Yes	70
Lee et al., 2022 [70]	Yes	Yes	Yes	Yes	Yes	Yes	No	60
Li et al., 2021 [71]	Yes	Yes	Yes	Yes	Yes	Yes	Yes	70
Majkowska et al., 2020 [72]	Yes	Yes	Yes	Yes	Yes	Yes	Yes	70
Mosquera et al., 2021 [73]	Yes	Yes	Yes	Yes	Yes	Yes	Yes	70
Nam et al., 2021 [74]	Yes	Yes	Yes	Yes	Yes	Yes	Yes	70
Niehues et al., 2021 [76]	Yes	Yes	Yes	Yes	Yes	Yes	Yes	70
Park et al., 2020 [77]	Yes	Yes	Yes	Yes	Yes	Yes	Yes	70
Paul et al., 2021 [78]	Yes	No	Yes	Yes	Yes	Yes	Yes	60
Pham et al., 2021 [80]	Yes	Yes	Yes	Yes	Yes	Yes	Yes	70
Rudolph et al., 2022 [81]	Yes	Yes	No	Yes	Yes	Yes	Yes	60
Rudolph et al., 2022 [82]	Yes	No	No	Yes	No	Yes	Yes	40
Seah et al., 2021 [83]	Yes	Yes	Yes	Yes	Yes	Yes	Yes	70
Senan et al., 2021 [84]	Yes	No	Yes	NA	Yes	Yes	Yes	60
Sharma et al., 2020 [85]	Yes	No	No	Yes	Yes	Yes	Yes	50
Sung et al., 2021 [87]	Yes	Yes	NA	NA	Yes	Yes	Yes	70
Van Beek et al., 2022 [88]	Yes	Yes	Yes	Yes	Yes	Yes	Yes	70
Verma et al., 2020 [89]	Yes	No	Yes	Yes	Yes	Yes	Yes	60
Wang et al., 2021 [90]	Yes	No	Yes	Yes	Yes	Yes	No	50
Wang et al., 2020 [91]	Yes	Yes	Yes	Yes	Yes	Yes	Yes	70
Wang et al., 2021 [92]	Yes	No	Yes	Yes	Yes	Yes	Yes	60
Wang et al., 2020 [93]	Yes	Yes	Yes	Yes	Yes	Yes	Yes	70
Wu et al., 2020 [11]	Yes	Yes	Yes	Yes	Yes	Yes	Yes	70
Xu et al., 2020 [94]	Yes	Yes	Yes	Yes	Yes	Yes	Yes	70
Zhou et al., 2021 [95]	Yes	Yes	Yes	Yes	Yes	Yes	Yes	70

**Table 4:** Summary of the published performance statistics of CXR deep learning models.

Deep Learning Model	Model Architecture	Model Validation Process	Model Performance and Study Results
Ahn et al., 2022 [43]	Not specified	Not specified—commercial	AI was associated with higher sensitivity for all findings compared with readers (nodule, 0.816 vs. 0.567; pneumonia, 0.887 vs. 0.673; pleural effusion, 0.872 vs. 0.889; pneumothorax, 0.988 vs. 0.792)
Albahli et al., 2021 [44]	Unet, NasNetLarge, Xception, Inception-V3, Inception-ResNetV2, ResNet50	Train, test	Test accuracy: 0.66 and 0.62
Altaf et al., 2021 [46]	DenseNet-201, ResNet50, Inception-V3, VGG-16	Train, test with cross-validation	Specificity 0.95, sensitivity 0.65, F1 0.53, accuracy 0.91
Baltruschat et al., 2021 [49]	ResNet-50	5-fold resampling scheme—7:1:2 (training, validation, testing)	Average RTAT for all critical findings was significantly reduced in all prioritization simulations compared to the first-in-first-out simulation, while the maximum RTAT for most findings increased. Pneumothorax (Min/Max) 80.1/890 vs. 35.6/1178, congestion 80.5/916 vs. 45.3/2018, pleural effusion 80.5/932 vs. 54.6/2144, infiltrate 80.3/916 vs. 59.1/2144, atelectasis 80.4/906 vs. 61.7/1958, cardiomegaly 80.5/932 vs. 62.5/1698, mass 81.0/902 vs. 64.3/1556, foreign object 80.4/930 vs. 80.6/2093, normal 80.2/940 vs. 113.9/2093
Bharati et al., 2020 [50]	VDSNet	Train, test	Accuracy 0.73
Chakravarty et al., 2020 [51]	DenseNet-121	Train, test	Average AUC 0.82
Chen et al., 2020 [53]	DenseNet-121	Train, test	Average AUC 0.89
Cho et al., 2020 [55]	eDenseYOLO	Train, tune, test (7:1:2)	Percent positive agreement: 83.39%, 74.14%, 95.12%, 96.84%, and 84.58%
Cho et al., 2020 [56]	ResNet-50	Train, tune, test (7:1:2)	Accuracy: 0.90, 0.90, 0.91, 0.92, and 0.93
Choi et al., 2021 [57]	Insight CXR, Lunit	-	Average AUC 0.99, sensitivity 0.97, specificity 0.93, and accuracy of 0.96. The model outperformed board-certified radiologists, non-radiology physicians, and general practitioners. Average AUC of physicians was 0.87 without model assistance and 0.91 with model assistance
Fang et al., 2021 [58]	CXR-IRNet	Train, test, validation	Average AUC 0.83
Gipson et al., 2022 [59]	EfficientNet architecture, segmentation CNN based on U-Net/EfficientNet backbone	Not specified	AI superior to radiologists for pneumothorax (AI AUC = 0.926, sens. = 39.2%, spec. = 99.8%, FP *n* = 2, *p* = 0.007) and lobar/segmental collapse (AI AUC = 0.917, sens. = 36.1%, spec. = 98.5%, FP *n* = 21, *p* = 0.012). AI inferior for clavicle (AI AUC = 0.831, sens. = 55.7%, spec. = 97.2%, FP *n* = 37, *p* = 0.002), humerus (AI AUC = 0.836, sens. = 32.3%, spec. = 99.4%, FP *n* = 8, *p* < 0.001), and scapular fracture (AI AUC = 0.855, sens. = 34.6%, spec. = 95.2%, FP *n* = 64, *p* = 0.014). No sig. diff. for rib fracture (AI AUC = 0.749, sens. = 41.1%, spec. = 92.9%, FP *n* = 75, k = 0.39) and pneumomediastinum (AUC = 0.872, sens. = 11.1%, spec. = 100%, FP *n* = 0, k = 0.19)
Gündel et al., 2021 [60]	DenseNet architecture	Train, test, validation	Average AUC 0.88
Han et al., 2022 [61]	ChexRadiNet	Not specified	The model achieved AUC scores of 0.831, 0.934, 0.817, 0.906, 0.892, 0.925, 0.798, 0.882, 0.734, 0.846, 0.748, 0.867, 0.737, and 0.889, respectively, for the pathologies (atelectasis, cardiomegaly, consolidation, edema, effusion, emphysema, fibrosis, hernia, infiltration, mass, nodule, pleural thickening, pneumonia, and pneumothorax)
Hwang et al., 2022 [62]	Not specified	Not specified—commercial	16.5% of scans initially labeled normal classified abnormal by model. 103/591 were clinically relevant (488 false positives). 13.3% of detected abnormalities accepted by radiologist. Situation (a) AI as the advisor: detection yield = 1.2%, FRR = 0.97%, PPV = 55.4%. Situation (b) AI as the final consultant: detection yield = 2.4%, FRR = 14%, PPV = 14.8%. Higher net benefit of AI as an advisor
Jabbour et al., 2022 [63]	CNN with DenseNet-121 architecture	External validation	Pneumonia: (k = 0.47) (AUC: combined int. = 0.71, ext. = 0.65) (Combined sens. = 81%, spec. = 60%), heart failure: (k = 0.48) (AUC: combined int. = 0.82, ext. = 0.82) (Combined sens. = 62%, spec. = 83%), COPD: (k = 0.56) (AUC: combined int. = 0.76, ext. = 0.86) (Combined sens. = 68%, spec. = 94%). Combined model sensitivity higher than both other models, lower specificity than both other models. Combined model AUROC higher than physician for heart failure (0.79 vs. 0.77) and COPD (0.89 vs. 0.78), lower for pneumonia (0.74 vs. 0.75)
Jadhav et al., 2020 [64]	VGGNet (16 layers), ResNet (50 layers)	Train, validation, test (7:1:2)	Precision 0.85, recall 0.83, F1 0.84
Jin et al., 2022 [66]	Not specified	Not specified—commercial	Standalone model performance average sensitivity, specificity, and AUC of 0.885, 0.723, and 0.867, respectively. For readers, average AUC and AUAFROC significantly increased with AI assistance (from 0.861 to 0.886; *p* = 0.003 and from 0.797 to 0.822; *p* = 0.003, respectively)
Jones et al., 2021 [42]	EfficientNet architecture, segmentation CNN based on U-Net/EfficientNet backbone	Not specified	90% of radiologists reported increased reporting accuracy with model by radiologists w/ (a) 5> yrs. experience, (b) 6–10 yrs. exp., (c) 10+ yrs. Exp., (a) 5% (b) 1.3% (c) 1.6% rate of sig. report change with model, (a) 2.4% (b) 0.4% (c) 0.9% patient management change, and (a) 1.5% (b) 0.5% (c) 0.6% increase in recommendations for further imaging. No sig. impact of radiologist experience on these rates
Kim et al., 2021 [67]	Insight CXR, Lunit	-	Sensitivity 0.83, specificity 0.79
Kim et al., 2022 [68]	ResNet-34 based architecture	Not specified—commercial	Thoracic abnormalities were found in 343 cases (11.0%) based on the CXR radiology reports and 621 (20.1%) based on the Lunit results. The concordance rate was 86.8% (accept: 85.3%, edit: 0.9%, and add: 0.6%), and the discordance rate was 13.2%. The median reading time increased after the clinical integration of Lunit (median, 19 s vs. 14 s, *p* < 0.001)
Kuo et al., 2021 [69]	-	Train, test, validation and external validation	Average AUC 0.75
Lee et al., 2022 [70]	DuETNet: DenseNet backbone, dual encoder	Train, validation, test	Model superior to all other models. AUC: atelectasis = 0.7711, cardiomegaly = 0.914, effusion = 0.8197, infiltration = 0.7096, mass = 0.8582, nodule = 0.8223, pneumonia = 0.8928, pneumothorax = 0.8805, consolidation = 0.7976, edema = 0.8892, emphysema = 0.9331, fibrosis = 0.93, PT = 0.8493, hernia = 0.997, average AUC = 0.8617
Li et al., 2021 [71]	ResNet-38	Train, test, validation with 10-fold cross-validation	The model generated statistically significant higher AUC performance compared with radiologists on atelectasis, mass, and nodule, with AUC values of 0.83, 0.96, and 0.93, respectively. For the other 11 pathologies, there were no statistically significant differences
Majkowska et al., 2020 [72]	-	Train, validation, test	AUCs 0.94, 0.91, 0.94, and 0.81
Mosquera et al., 2021 [73]	RetinaNet, Inception-ResnetV2, AlbuNet-34	Train, test, external validation	AUCs 0.75 and 0.87, sensitivity 0.86, specificity 0.88
Nam et al., 2021 [74]	ResNet34	-	Model AUCs 0.90–1.00 (CT-confirmed dataset) and 0.91–1.00 (PadChest dataset). The model correctly classified significantly more critical abnormalities (95%) than radiologists (84%). Radiologists detected significantly more critical (71% vs. 29%) and urgent (83% vs. 78%) abnormalities when aided by the model
Niehues et al., 2021 [76]	-	Train, test	AUCs 0.90, 0.95, 0.85, 0.92, 0.99, 0.99, 0.98, and 0.99
Park et al., 2020 [77]	-	Train, validation, test	AUC 0.99 vs. 0.96
Paul et al., 2021 [78]	DenseNet	Train, test, external validation	AUCs 0.55–0.79
Pham et al., 2021 [80]	-	Train, validation, test	Average AUC 0.94 (validation set), 0.93 (test set)
Rudolph et al., 2022 [81]	Not specified	External validation	AUC of 0.940 (pneumothorax), 0.953 (pleural effusion), 0.883 (lung lesions), and 0.847 (consolidation). The AI system matched radiology residents’ performance, and significantly outperformed non-radiology residents’ diagnostic accuracy for pneumothorax, pleural effusion, and lung lesions
Rudolph et al., 2022 [82]	CheXNet	External validation	CheXNet was similar to radiology resident (RR) detection of suspicious lung nodules (cohort, AUC AI/RR: 0.851/0.839, *p* = 0.793), basal pneumonia (cohort, AUC AI/reader consensus: 0.825/0.782, *p* = 0.390), and basal pleural effusion (cohort, AUC AI/reader consensus: 0.762/0.710, *p* = 0.336)
Seah et al., 2021 [83]	EfficientNet	Train, test	Average AUC of the model 0.96. Average AUC of unassisted radiologists 0.72. Average AUC when radiologists used the model 0.81. Model use significantly improved accuracy for 102 (80%) clinical findings
Senan et al., 2021 [84]	ResNet-50 and AlexNet	Not specified	The ResNet-50 network reached average accuracy, sensitivity, specificity, and AUC of 95%, 94.5%, 98%, and 97.10%, respectively
Sharma et al., 2020 [85]	Not reported	Train, test, external validation	Accuracies 1.00, 1.00, 0.95, 0.00, 0.94, and 0.00
Sung et al., 2021 [87]	Med-Chest X-ray	-	AUC of radiologists using the model (from 0.93 to 0.98), sensitivity (from 0.83 to 0.89), and specificity (from 0.89 to 0.97)
Van Beek et al., 2022 [88]	ResNet34 basis, AutoAugment + Attend and Compare modules, binary cross-entropy loss function	Not specified—commercial	Atelectasis (AUC = 0.914, 0.891, sens. = 0.816, 0.55, spec. = 0.887, 0.961), calcification (AUC = 0.92, 0.922, sens. = 0.765, 0.692, spec. = 0.887, 0.919), cardiomegaly (AUC = 0.943, 0.97, sens. = 0.88, 0.85, spec. = 0.884, 0.962), consolidation (AUC = 0.903, 0.881, sens. = 0.886, 0.922, spec. = 0.792, 0.674), fibrosis (AUC = 0.948, 0.92, sens. = 0.933, 0.714, spec. = 0.895, 0.924), mediastinal widening (AUC = 0.909, 0.998, sens. = 0.8, 1, spec. = 0.97, 0.993), nodule (AUC = 0.881, 0.905, sens. = 0.794, 0.833, spec. = 0.848, 0.886), pleural effusion (AUC = 0.954, 0.988, sens. = 0.784, 0.837, spec. = 0.942, 0.986), pneumoperitoneum (AUC = 0.999, insuff. case no., sens. = 1, --, spec. = 0.975, 0.996), pneumothorax (AUC = 0.954, insuff. case no., sens. = 0.833, 1, spec. = 0.978, 0.992). Non-significant difference of performance in acute and non-acute sources; model outperformed radiologists for all findings
Verma et al., 2020 [89]	Not reported	Train, test	Accuracy 0.99
Wang et al., 2021 [90]	MARnet	5-fold cross-validation	AUC: nodule 0.90, atelectasis 0.93, normal 0.99, infection 1.00. MARnet outperformed all other CNNs
Wang et al., 2020 [91]	Thorax-Net	Train, test, validation	Average AUC 0.79 and 0.90
Wang et al., 2021 [92]	DenseNet-121	Train, test, validation	Average AUC 0.83
Wang et al., 2020 [93]	DenseNet-121	Train, validation	Average AUC 0.82
Wu et al., 2020 [11]	ResNet50, HVGG16 S	Train, validation, test	Average AUC: model 0.77, residents 0.72. PPV: model 0.73, residents 0.68. Specificity: model 0.98, residents 0.97
Xu et al., 2020 [94]	MS-ANet	Train, test, validation	Average AUCs 0.85 and 0.82
Zhou et al., 2021 [95]	-	Training, test (9:1)	Accuracy for cardiomegaly 0.98, pneumothorax 0.71, and pleural effusion 0.78

**Table 5:** Summary of the clinical benefits of CXR machine learning systems identified in the literature.

Identified Benefit	Clinical Setting	Associated Clinical Outcomes	Reference
Improved radiologist accuracy in detecting pathology on the medical image	All clinical settings	Reduced unnecessary and increased appropriate follow-up CT examinations or earlier detection of findings, leading to improved patient outcomes. Reduction in false positives	Choi et al., 2021 [57] Nam et al., 2021 [74] Seah et al., 2021 [83] Sung et al., 2021 [87] Jin et al., 2022 [66] Jones et al., 2021 [42] Hwang et al., 2022 [62] Ahn et al., 2022 [43]
Reduced time to report studies that contain critical pathology	All clinical settings	Reduction in report turnaround time for sensitive findings such as pneumothorax and rib fracture allowing correct patient management and earlier treatment	Nam et al., 2021 [74] Baltruschat et al., 2021 [49]
Reduced per-study reporting time	Inpatient or outpatient or screening	Increased reporting efficiency	Nam et al., 2021 [74] Sung et al., 2021 [87] Ahn et al., 2022 [43]
Consistent detection accuracy across variations in image quality	Inpatient or outpatient or screening or emergency	Accurate detection of pathology on CXRs regardless of imaging source or quality of the acquisition	Choi et al., 2021 [57] Gündel et al., 2021 [60] Rudolph et al., 2022 [82] van Beek et al., 2022 [88]

## Data Availability

The datasets used and/or analyzed during the current study are available from the corresponding author on request.

## Appendix A. Assessment Criteria for Quality and Risk of Bias

**Table A1:** Risk of bias assessment of included studies. + Indicates low ROB, - indicates high ROB, and ? indicates unclear ROB.

PROBAST ROB Assessment					
Study	Participants	Predictors	Outcome	Analysis	Overall
Ahn et al., 2022 [43]	+	+	+	+	+
Albahli et al., 2021 [44]	+	+	+	+	+
Altaf et al., 2021 [46]	+	+	+	+	+
Baltruschat et al., 2021 [49]	+	+	+	+	+
Bharati et al., 2020 [50]	+	+	+	+	+
Chakravarty et al., 2020 [51]	+	+	+	+	+
Chen et al., 2020 [53]	+	+	+	+	+
Cho, Kim et al., 2020 [55]	+	+	+	+	+
Cho, Park et al., 2020 [56]	+	+	+	+	+
Choi et al., 2021 [57]	+	+	+	+	+
Fang et al., 2021 [58]	+	+	+	+	+
Gipson et al., 2022 [59]	+	+	+	+	+
Gündel et al., 2021 [60]	+	+	+	+	+
Han et al., 2022 [61]	+	+	+	+	+
Hwang et al., 2022 [62]	+	+	+	+	+
Jabbour et al., 2022 [63]	+	+	-	+	-
Jadhav et al., 2020 [64]	+	+	+	+	+
Jin et al., 2022 [66]	+	+	+	+	+
Jones et al., 2021 [42]	+	+	-	+	+
Kim et al., 2021 [67]	+	+	+	+	+
Kim et al., 2022 [68]	+	+	+	+	+
Kuo et al., 2021 [69]	+	+	+	+	+
Lee et al., 2022 [70]	+	+	+	+	+
Li et al., 2021 [71]	+	+	+	+	+
Majkowska et al., 2020 [72]	+	+	+	+	+
Mosquera et al., 2021 [73]	+	+	+	+	+
Nam et al., 2021 [74]	+	+	+	+	+
Niehues et al., 2021 [76]	+	+	+	+	+
Park et al., 2020 [77]	+	+	+	+	+
Paul et al., 2021 [78]	+	+	+	+	+
Pham et al., 2021 [80]	+	+	+	+	+
Rudolph et al., 2022 [81]	-	+	+	+	-
Rudolph et al., 2022 [82]	-	+	+	+	-
Seah et al., 2021 [83]	+	+	+	+	+
Senan et al., 2021 [84]	-	+	+	+	-
Sharma et al., 2020 [85]	+	+	+	-	-
Sung et al., 2021 [87]	+	+	+	+	+
Van Beek et al., 2022 [88]	+	+	+	+	+
Verma et al., 2020 [89]	+	+	+	?	?
Wang et al., 2021 [90]	-	+	+	+	-
Wang et al., 2020 [91]	+	+	+	+	+
Wang et al., 2021 [92]	+	+	+	+	+
Wang et al., 2020 [93]	+	+	+	+	+
Wu et al., 2020 [11]	+	+	+	+	+
Xu et al., 2020 [94]	+	+	+	+	+
Zhou et al., 2021 [95]	+	+	+	+	+
